# Delphi Method Exploration of Dementia Program Metrics: Preliminary Insights from Expert Panel

**DOI:** 10.1002/alz.093154

**Published:** 2025-01-09

**Authors:** Shadi Gholizadeh, Matthew Neal, Kirk Hayes, John Mark Geiss

**Affiliations:** ^1^ Brigade Health, Orange, CA USA; ^2^ Senior Doc, Orange, CA USA

## Abstract

**Background:**

Measuring the effectiveness of dementia care programs is essential for ensuring quality care and aligning with value‐based care principles, especially in practical, real‐world clinical settings. A Delphi method, a consensus‐building approach among experts, was used to identify practical metrics for evaluating dementia programs. The expert panel comprised a clinical psychologist, two healthcare executives, and a physician experienced in person‐centered care for older adults in residential and facility settings.

**Method:**

The Delphi method involved a series of structured questionnaires sent to the panelists. The process began with open‐ended queries regarding crucial outcomes and indicators for dementia care effectiveness. Through iterative rounds, the panel’s collective input was used to hone in on the most relevant and practical metrics for real‐world clinical assessment of dementia programs.

**Result:**

The expert panel reached a consensus on several key metrics, including patient and caregiver satisfaction, efficacy in managing behavioral symptoms, reduction in emergency room visits and hospital admissions, and medication adherence. Additional metrics included health behaviors among patients and caregivers, caregiver perceptions of burden and resilience, and the actual engagement with and utilization of recommended caregiver support and education. A notable discussion point was the mode and frequency of assessing these metrics, with a recognition of the need for parsimony to minimize patient and caregiver burden and practicality and ease of assessment and scoring for non‐clinical medical staff.

**Conclusion:**

This Delphi study provided critical insights into relevant metrics for dementia program evaluation. The identified preliminary metrics serve as a foundation for further refinement with a broader expert panel, including considerations for assessment methods and frequency. The ultimate aim is to develop a comprehensive, practical set of metrics that accurately reflects dementia care program quality and impact, catering to the needs of smaller clinical practices. These measures will support continuous improvement and adherence to value‐based care goals, enhancing dementia care quality across diverse clinical settings.

